# NCCN Imaging Appropriate Use Criteria Compendium: An Overview

**DOI:** 10.6004/jadpro.2017.8.2.8

**Published:** 2017-03-01

**Authors:** Mistie G. Hagaman

**Affiliations:** Wellmont Cancer Institute, Norton, Virginia

The National Comprehensive Cancer Network™ (NCCN™) website has an evidence-based tool called the NCCN Imaging Appropriate Use Criteria (AUC) Compendium™ to help guide practitioners in choosing appropriate imaging for their patients. The NCCN Imaging AUC Compendium was developed in early 2016. The tool supports the most recent versions of the NCCN Guidelines™, with at least annual review.

The NCCN Imaging AUC Compendium is a quick, easy-to-use tool to guide imaging decisions in the care plan of an individual with cancer, whether in screening, diagnosis, staging, treatment response assessment, or surveillance. The tool uses 44 diagnoses and references the NCCN Guidelines™ and page number where the information can be found. The design for decision-making on appropriate imaging, support, and timing in the NCCN Imaging AUC Compendium is developed by the multidisciplinary NCCN Guidelines panels, which include physicians, nurses, nurse practitioners, radiologists, and pharmacists (NCCN Guidelines, 2016). Like the NCCN Guidelines, these criteria are developed from the most recent scientific evidence, expert panel recommendations, and published literature.

## ACCESS

The NCCN Imaging AUC Compendium can be accessed on the NCCN website at http://www.nccn.org. From the main page, click on the NCCN Compendia tab at the top and then click on the fourth option down: NCCN Imaging AUC Compendium. Next, slide right to search the NCCN Imaging AUC Compendium. Before accessing this free tool, you will need to create an account by providing an e-mail address and accepting an End-User License Agreement.

## NAVIGATION AND USE

The NCCN Imaging AUC Compendium tool consists of user-friendly, drop-down menus to guide the user specifically through categories associated with certain NCCN-supported guidelines. The tool may be customized by selecting the fields to display or hide. The drop-down menus allow the choice of a specific guideline, clinical setting (more specific diagnosis), purpose (such as diagnosis or staging), modality (type of test), and/or ICD-10 code.

For example, the option of Breast Cancer within the NCCN Guideline drop-down menu will display only breast cancer–related diagnoses in the Clinical Setting drop-down menu vs. using the Prostate Cancer option and then having only the Prostate Cancer option under the Clinical Setting drop-down menu. The Clinical Setting allows a more specific choice, narrowing the image decision-making options.

Under the Purpose drop-down menu, there are four options: Diagnostic/Staging, Treatment Response Assessment, Screening, and Follow-up/Surveillance (NCCN Imaging, 2016). These options can vary based on the selected cancer or screening guideline. Under Modality, options range from bone scans, magnetic resonance imaging, positron-emission tomography/computed tomography (CT), mammography, CT scans, ultrasounds, and simple x-rays (listed in alphabetical order). The information can be filtered to show only fields the user would like to see. Results are listed in descending order by guideline page number; however, the order may be customized to the user’s preference, and the results are printable. The information provided by this tool includes the indication for the scan, what type of scan is most appropriate, and how frequently the scan should be ordered.

In addition to the great benefits of the tool, ICD-10 codes are added for provider convenience. For added simplicity, there is an NCCN Imaging AUC Compendium User Guide on the page prior to accessing the tool.

Advanced practitioners will find this tool useful in guiding decisions regarding scan choices. Data from this tool will provide needed evidence-based documentation to justify scans for payers.

## SUMMARY

The NCCN Imaging AUC Compendium uses the data compiled by NCCN panel members in a convenient, easy-to-use tool to help guide practitioners order the imaging and surveillance deemed most appropriate and cost-effective. The imaging tool is a quick and informative reference tool. As knowledge increases and changes, so will the guidelines and tools. Additional cancers are being added quarterly; in 2016, 44 cancers were added, with 2 more scheduled to be added in 2017.

**Permission**

NCCN referenced with permission from the NCCN Imaging Appropriate Use Criteria Compendium™ (NCCN Imaging AUC Compendium™) © National Comprehensive Cancer Network, Inc 2017. All rights reserved. To view the most recent and complete version of the NCCN Imaging AUC Compendium™, go online to NCCN.org. National Comprehensive Cancer Network®, NCCN®, NCCN Guidelines®, and NCCN Imaging AUC Compendium™, and all other NCCN Content are trademarks owned by the National Comprehensive Cancer Network, Inc.
